# A rare case of dermoid cyst in the pancreas

**DOI:** 10.1093/bjrcr/uaaf031

**Published:** 2025-06-10

**Authors:** Syer Ree Tee, Zainab Al Manji, Donal Maguire, Niall Swan, Sinead H McEvoy

**Affiliations:** Department of Radiology, St. Vincent’s University Hospital, Dublin, D04 T6F4, Ireland; Department of Histopathology, St. Vincent’s University Hospital, Dublin, D04 T6F4, Ireland; Department of Surgery, St. Vincent’s University Hospital, Dublin, D04 T6F4, Ireland; Department of Histopathology, St. Vincent’s University Hospital, Dublin, D04 T6F4, Ireland; Department of Radiology, St. Vincent’s University Hospital, Dublin, D04 T6F4, Ireland

**Keywords:** pancreatic dermoid cyst, mature cystic teratoma, cystic neoplasm of the pancreas

## Abstract

Pancreatic dermoid cyst is an extremely rare benign neoplasm of the pancreas. Pre-operative diagnosis is often difficult due to its rarity and overlapping features with other pancreatic cystic neoplasms. We report a case of a 55-year-old male with an incidental finding of a lobulated complex cystic lesion in the tail of the pancreas on imaging and the challenges to obtain a definitive diagnosis. Due to suspicious features on imaging and elevated CA 19-9 tumour marker, surgical resection was recommended at the Pancreatic Multidisciplinary Team Meeting. The patient subsequently underwent a distal pancreatectomy and splenectomy which confirmed a dermoid cyst.

## Background

Mature cystic teratoma of the pancreas is an extremely rare benign neoplasm of the pancreas. It is of germ cell origin derived from totipotent stem cells with the ability to generate tissues from all 3 germ layers: endodermal, mesodermal, and ectodermal. Mature cystic teratoma with predominance of ectodermal tissue differentiation is also known as dermoid cyst.

The most common site of presentation is the ovaries. However, they may be encountered anywhere along the midline of the body following the route of germ cell migration during embryogenesis and can present in the mediastinum, retroperitoneum, and sacrococcygeal region. The pancreas is an extremely rare site of presentation, with approximately 50 cases published in the literature. Pre-operative diagnosis is challenging due to its rarity and overlapping features with other pancreatic cystic neoplasms.

In this report, we present a rare case of pancreatic dermoid cyst from our institution.

## Case

A 55-year-old male with a background history of Type 2 diabetes mellitus, hypertension, and dyslipidaemia presented to a peripheral hospital with exertional shortness of breath and palpitations since COVID infection several months prior. Physical examination was normal apart from high body mass index. Blood investigations showed a low mean cell volume of 70 fL (normal values 80-100 fL) with normal haemoglobin and a slightly elevated D-Dimer of 0.9 ug FEU/mL (normal values 0-0.5 ug FEU/mL) which prompted a CT pulmonary angiogram (CTPA) to further investigate. CTPA showed no pulmonary embolism but there was an incidental finding of a lobulated cystic mass in the tail of the pancreas. The patient was asymptomatic from this.

**Figure 1.**
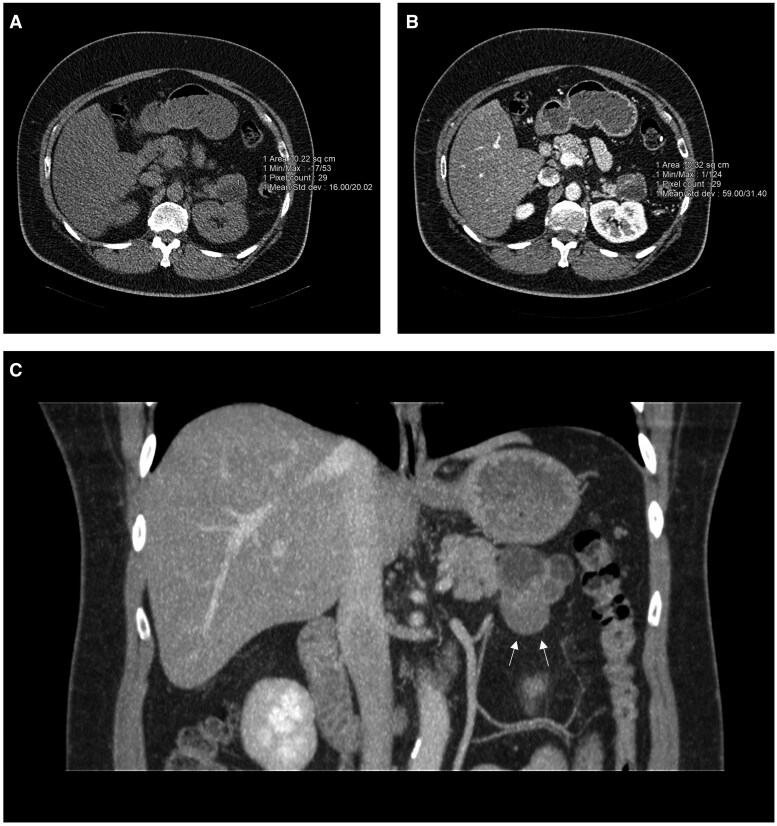
Computed tomography images showing a lobulated septated cystic mass in the tail of the pancreas. (A) Axial non-contrast and (B) arterial phase sequences demonstrating enhancement of the anterior solid component. (C) Coronal view of the mass in arterial phase (white arrows).

Subsequent CT pancreas (Figure 1) showed a 4.4 × 3.8 × 5.0 cm lobulated complex cystic mass in the tail of pancreas. While predominantly of fluid attenuation, there was an enhancing nondependent solid mural component in the anterior aspect of the mass. No internal or mural calcification or focal fat. No communication with the main pancreatic duct was demonstrated. No evidence of lymphadenopathy or other worrisome extrapancreatic abnormality. Magnetic Resonance Cholangiopancreatography (MRCP) and MRI pancreas were not performed as there was no MRI facility at the hospital where the patient attended.

Further blood investigation with tumour markers showed an elevated CA 19-9 of 90.0 kU/L (normal limits 0-37 kU/L). Alpha-fetoprotein, carcinoembryonic antigen (CEA), and CA 15-3 markers were within normal limits.

The patient’s case was then referred to our institution which is the National Surgical Centre for Pancreatic Cancer (NSCPC) for discussion at the weekly Multidisciplinary Team (MDT) Meeting.

Given the imaging findings, the main differential diagnoses were cystic pancreatic neuroendocrine tumour (pNET) or transformed side-branch intraductal papillary mucinous neoplasm (IPMN). Cystic pNET was considered due to its cystic component, enhancement, well-circumscribed fibrous capsule and its location in the pancreatic tail. Cystic pNETs are more commonly located in the neck, body, or tail of the pancreas than in the head compared with solid counterpart.^1^ Transformed side-branch IPMN was considered due to its multilobulated appearance, presence of enhancing solid component with a raised CA 19-9. Communication with the main pancreatic duct was not seen on CT imaging; however, this would have been better assessed on MRCP; an IPMN would demonstrate communication with the pancreatic duct whereas a pNET does not communicate with the pancreatic duct.

Solid and papillary epithelial neoplasm (SPEN) was considered due to its imaging appearance of an encapsulated mass with cystic and solid components, however, considered less likely due to demographics. SPEN occurs predominantly in young women with mean age of 35 years.^2^ Mucinous cystic neoplasm, almost exclusively seen in females was also considered less likely as the patient is male. Appearances were not suggestive of serous cystic neoplasm (SCN) which usually demonstrates cluster of microcysts (usually more than 6) that range from a few millimetres up to 2 cm in size. A fibrous central scar with stellate calcification on CT, seen in 30% of SCN is considered highly specific and pathognomonic.^2^ Pancreatic pseudocyst was also considered less likely due to lack of precipitating cause such as prior pancreatitis or trauma.

To best differentiate between the 2 main differential diagnoses of cystic pNET and transformed side-branch IPMN, MDT decision was to perform a Gallium DOTA-TOC PET-CT. An MRCP, MRI pancreas, endoscopic ultrausound (EUS), and fine needle aspiration (FNA) were also recommended.

**Figure 2.**
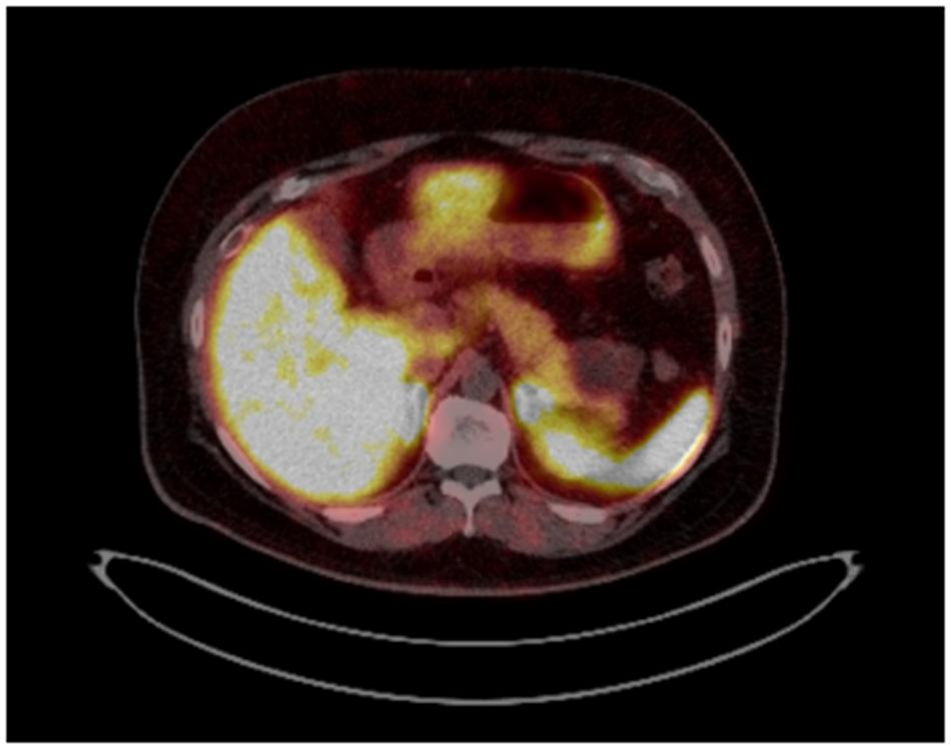
Gallium PET-CT performed showed no somatostatin receptor avidity in the pancreatic lesion.

The patient subsequently underwent Gallium DOTA-TOC PET-CT (Figure 2) to further evaluate. No somatostatin receptor avidity was identified in the pancreatic lesion. The patient was scheduled for EUS but unfortunately did not attend as he was unwell.

The patient’s case was rediscussed at the pancreatic MDT following Gallium DOTA-TOC PET-CT findings. Lack of somatostatin receptor avidity on the PET-CT makes the diagnosis of cystic pNET less likely. MRCP and MRI pancreas were scheduled but due to the long outpatient MRI wait list, was not performed prior to the MDT discussion. Due to concerning imaging features (size and enhancing solid component) and elevated CA 19-9, surgical resection was recommended.

The patient subsequently underwent an exploratory laparotomy, distal pancreatectomy, and splenectomy. The patient had an uneventful post-operative recovery and subsequently discharged home on post-operative day 9. There is lack of follow-up unfortunately as the patient was shortly readmitted to a peripheral hospital nearer to his home upon discharge with respiratory sepsis and had a prolonged inpatient stay. He did not attend subsequent follow-up outpatient appointments.

**Figure 3.**
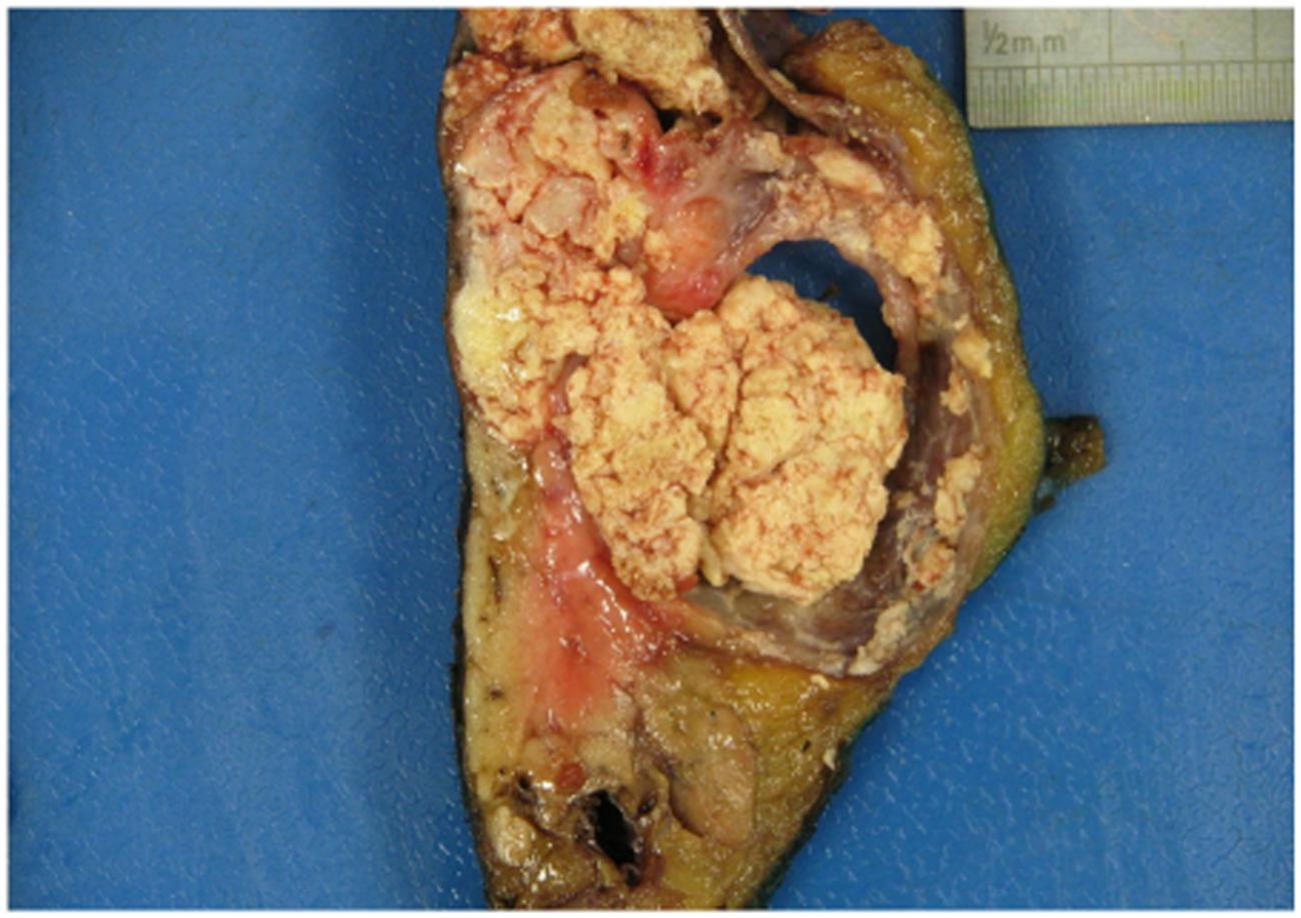
Tail of pancreas with a cystic lesion filled with sebaceous material.

**Figure 4.**
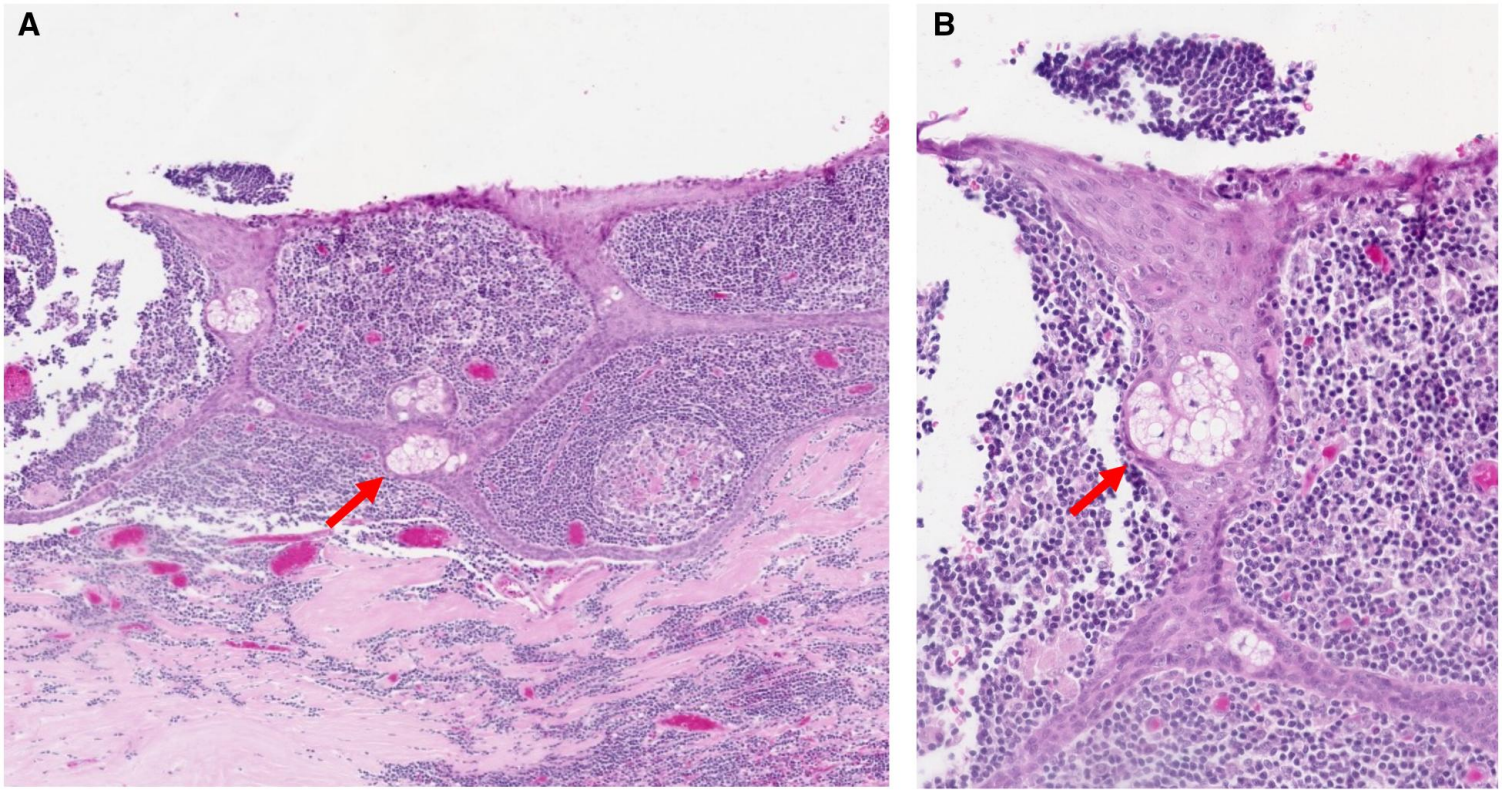
(A) and (B) Haematoxylin and eosin stain of a dermoid cyst lined by stratified squamous epithelium with cutaneous sebaceous glands (red arrow) and prominent underlying lymphoid stroma.

Macroscopic examination of the excised distal pancreas revealed a well-circumscribed intrapancreatic cystic lesion with intraluminal sebaceous content, measuring 6.5 × 8.4 × 3.2 cm (Figure 3). Microscopically, the cyst was lined by mature keratinizing squamous epithelium with underlying prominent lymphoid stroma and cutaneous adnexal structures (Figure 4A and B). The lumen of the cyst contained abundant keratinous debris corresponding to the sebaceous content observed macroscopically. There was no evidence of malignancy. The background pancreatic parenchyma was unremarkable.

## Discussion

Dermoid cysts are germ cell originated, benign, slow-growing tumours composed of mature differentiated elements derived from multiple embryonic layers. It predominantly occurs in the ovaries, testes, mediastinum, and retroperitoneum. The pancreas is an extremely uncommon primary location, with only approximately 50 cases reported in the literature.^3^

In terms of clinical features, there is a slight male predominance^4^^,^^5^ and occurrence in a slightly younger age group, ranging from 2 to 70 years.^4^ The size of dermoid cysts reported in the literature varies (2.2-25 cm with an average size of 8 cm).^3^ They tend to be slow growing and tend to present when relatively large. They arise mostly from the body of the pancreas, accounting for 35.6% of cases followed by the head at 33.3%. The tail is involved in 15.5% of cases.^6^ Although patients with pancreatic dermoid cysts are generally asymptomatic, they can present with non-specific gastrointestinal symptoms such as diffuse abdominal pain, dyspepsia, nausea, or back pain.^4^ Pancreatitis due to dermoid cyst is an uncommon presentation.^7^

Physical examination is frequently normal, however, larger lesions may result in a palpable mass on exam. There are no specific laboratory examinations and tumour markers are generally within normal limits. Biochemical markers like CA 19-9 and CEA used for diagnosis of cystic pancreatic neoplasms are usually not elevated in patients with dermoid cysts. However, CA 19-9 could be elevated, complicating the differential diagnosis and raising the suspicion for malignancy.^8^

In terms of imaging, the radiological appearance of dermoid cysts depends on its tissue contents such as proportion of fat and calcifications.^9^ On ultrasound, they appear as well-defined hyperechoic masses with calcific foci causing acoustic shadowing. On CT, they appear as well-defined, fat-containing lesions and may present with fat-fluid level and calcification in their walls. MRI is useful for distinguishing soft tissue components or to confirm presence of fat. Dermoid cysts do not communicate with the main pancreatic duct. Imaging findings however, is not always pathognomonic and overlaps with other pancreatic cystic lesions, both benign and malignant such as mucinous cystadenomas, pseudocysts, serous cystadenomas, lymphoepithelial cysts, and epidermoid cysts. Furthermore, the presence of fat fluid or hair fluid levels typically present in dermoid cysts in other locations are only present in a minority of cases^9^ and weren’t present in our case.

Endoscopic ultrasonography (EUS) with FNA is an important part of the evaluation of pancreatic cystic lesions and is used to sample the cyst wall and its contents for cytological and biochemical analysis.^10^ Whitish, necrotic material on FNA may give the impression of sebaceous secretions. Cytology may show benign appearing, mature squamous cells, inflammatory cells, and keratin debris. However, FNA cytology of lymphoepithelial cysts may yield similar findings, complicating the diagnosis.^11^

Examination of an excision specimen is usually required for a definitive diagnosis, which will demonstrate a benign mature squamous epithelial lining with underlying cutaneous adnexal structures (sebaceous glands and hair follicles). An associated prominent lymphoid stroma is usually present but the presence of skin adnexae aids in differentiating a dermoid cyst from a lymphoepithelial cyst, which is the main histological differential diagnosis.^4^ Malignant transformation of retroperitoneal teratomas is extremely uncommon and represents only 1%-2% of cases.^12^ Histopathological evaluation of the entire cyst wall is essential to rule out the presence of any immature foci.

Treatment options for dermoid cysts described in the literature^4^ include various drainage and resection techniques such as external and internal drainage (cystogastrostomy), simple cyst enucleation, and pancreatic resection. External drainage is associated with high recurrence and complication rates, particularly pancreatic fistula and is now generally not undertaken.^3^^,^^10^ There is limited evidence with regards to internal drainage. Simple cyst enucleation or cystectomy was associated with high reoperation rate. Surgical excision, either via open or laparoscopic approach is currently the preferred treatment of choice for all pancreatic dermoid cysts, either cystectomy or more radical resections depending on the size of the lesion, its adhesions and its anatomic relations with other organs. Recently, robotic-assisted resection of a dermoid cyst^13^ has been described as a new technique in the literature.

## Conclusion

Pancreatic dermoid cyst is a rare neoplasm in the pancreas with a difficult pre-operative diagnosis due to its rarity and overlapping features with other pancreatic cystic lesions. Surgical excision is usually required for definitive diagnosis and remains the standard of treatment for these lesions.

## Learning points

- Dermoid cysts are very rare cystic neoplasm of the pancreas.
- Imaging findings are not always pathognomonic and typical features such as presence of fat or fat fluid levels are not always present.
- Surgical excision is often required for definitive diagnosis.
